# Impact of Positive Childhood Experiences (PCEs): A Systematic Review of Longitudinal Studies

**DOI:** 10.1007/s10578-024-01807-x

**Published:** 2025-01-06

**Authors:** Kannan Kallapiran, Shuichi Suetani, Vanessa Cobham, Valsamma Eapen, James Scott

**Affiliations:** 1 Child Health Research Centre, The University of Queensland, South Brisbane, QLD 4101 Australia; 2 University of Queensland, St. Lucia, Australia; 3 Child and Youth Mental Health Service, Children’s Health Queensland Hospital & Health Service, South Brisbane, Australia; 4 Physical and Mental Health Research Stream, Queensland Centre for Mental Health Research, The Park Centre for Mental Health, Wacol, Australia; 5 School of Medicine and Dentistry, Griffith University, Southport, Australia; 6 University of New South Wales, Kensington, Australia; 7 Child and Youth Mental Health, Queensland Centre for Mental Health Research, The Park Centre for Mental Health, Wacol, Australia

**Keywords:** Resilience, Prevention, Promotion, Support

## Abstract

Positive Childhood Experiences (PCEs) may mitigate the negative outcomes resulting from Adverse Childhood Experiences (ACEs). To date, most PCE research has used cross-sectional or retrospective designs. PubMed, EMBASE, Cochrane, PsychINFO, CINAHL, and Scopus were searched in May 2024 for longitudinal studies that examined the impact of cumulative PCEs. Eight publications from five longitudinal studies with a total of 16,451 participants were included. Three studies focused only on adolescent outcomes. PCEs were associated with reduced rates of depression, substance use, delinquent behavior, risky sexual behavior, persistent insomnia, and lower inflammatory markers. Cumulative PCEs in childhood may moderate the impact of ACEs, potentially playing an important role in reducing the risk for mental disorders and other adverse outcomes later in life.

PROSPERO (ID: CRD42022384775).

**Supplementary Information:**

The online version contains supplementary material available at https://doi.org/10.1007/s10578-024-01807-x.

## Introduction

The landmark study by the Center for Disease Control (CDC)—Kaiser Permanente showed how cumulative scores of a list of Adverse Childhood Experiences (ACEs) were associated with many leading causes of death in adulthood [1] and these findings have been widely replicated [2]. Although the poor long-term outcomes associated with individual ACEs were previously known, the strong correlation between the cumulative scores of these interrelated adversities and a whole range of health outcomes has highlighted their salience in public health [3]. Increased awareness of cumulative ACEs has led to the development of prevention frameworks such as Preventing Adverse Childhood Experiences: Leveraging the Best Available Evidence [4]. Studies have documented that these frameworks can lead to substantial reductions in ACEs, which in turn promote broad and sustained benefits [5]. Despite the effectiveness of these frameworks, it is not always possible to prevent ACEs in practice. Further, the framework lacks consideration of protective childhood experiences and resilience factors that might buffer the long-term health effects of adverse experiences [6].

### Cumulative Positive Childhood Experiences (PCEs)

Positive Childhood Experiences (PCEs) have been described as safe, stable, nurturing relationships and environments. They form an important subset of resilience factors that could moderate the impact of ACEs [7]. Influenced by ACEs research, there has been an increasing interest in developing a PCE framework to inform cumulative experiences that could moderate the impact of ACEs [8, 9]. However, currently, there is no consensus on what factors should be included in such a PCE framework. Table 1 lists the four emerging dominant conceptual frameworks and the accompanying measures for PCEs.

**Table 1:** Frameworks and accompanying measures for Positive Childhood Experiences (PCEs)

Number	Framework	Description	Domains	Measures
1	Protective and Compensatory Experiences (PACEs)	Derived from research on resilience, neuroscience, epidemiology, child development and dynamic systems model. Modifiable or external factors such as relationships that can be strengthened and resources that can be provided rather than innate characteristics were included [10, 11]	Relationships: 1) Parent 2) Friendship 3) Community volunteering 4) Social groups 5) Adult outside of family Resources: 1) Safe home 2) Resources to learn 3) Hobbies 4) Engagement in sports 5) Family routine & consistent rules	PACEs Questionnaire: Retrospective measure Reliability: Internal consistencies Cronbach alpha ranged from 0 .70 to 0 .81 across different racial and ethnic groups Predictive validity: Retrospective PACEs score by parents correlated with nurturing parenting attitudes and moderated the impact of ACEs on harsh parenting [11]
2	Benevolent Childhood Experiences (BCEs)	Developed primarily based on knowledge from developmental psychopathology and ecological systems theory to describe positive experiences across multiple interacting systems [12, 13]	Items related to oneself (e.g. positive view of oneself, system of beliefs), one’s family (e.g. effective caregiver, predictable family routine) and a sense of connection to social community (e.g. a good friend, caring teacher, an effective school or a safe neighborhood)	BCEs scale: Retrospective measure Reliability: Test–retest reliability (r = 0.80, p < .01[14] Predictive validity: Higher BCEs significantly predicted lower PTSD symptoms Above and beyond the effects of ACEs (β = − 0.24, p < 0.05)
3	Bethell–Positive Childhood Experience items	Derived from the Child and Youth Resilience Measure- 28 item scale.7 key items derived from the 2015 Wisconsin Behavioral Risk Factor Survey that elicited information about safe, stable nurturing relationships and environment	Family, community traditions, belonging in high school, friendships, at least 2 supportive nonparent adults, safe protective parent or adult at home	Bethell – PCE items: Retrospective measure Reliability: Internal Consistency Cronbach’s alpha 0.77 Validity: 1) Construct validity: Factor loading: Two factors i) felt safe/ home and ii) family stood by/difficult times. Principal Component Analysis A single Eigen value greater than 1.0 (2.95) that explained 42.2% of variance 2) Predictive validity: Higher cumulative PCE scores predicted 72% lower odds for depression
4	HOPE Framework	Health Outcomes from Positive Experiences Framework (HOPE) comprises four core components [6]	A total of 20 examples (five for each component) 1) Nurturing and supportive relationships 2) Safe and protective environments 3) Constructive social engagement and connectedness 4) learning social and emotional competencies were provided. [6]	Modified HOPE framework: Prospective measure Reliability ^a^: Good internal coherence Validity ^a^: 1) Confirmatory Factor Analysis indicated good construct validity 2) Predictive validity: Cumulative PCE scores correlated with lower mental health problems and academic difficulties [15]

^a^Modified HOPE framework without social & emotional competencies for Longitudinal Study of Australian Children

The existing frameworks all emphasize that PCEs are not just the absence of ACEs but are positive experiences, assets, or resources that provide the necessary foundation for overall development. They agree that the PCEs occur at multiple levels of a child’s environment (family, school, neighborhood, and community) in line with Bronfenbrenner’s ecological theory and highlight the interrelatedness of those domains [16]. This signifies the importance of cumulative measures to prevent overestimation of the impact of individual factors. While all include safe, stable, nurturing relationships and environments in their framework [8], others additionally include resources such as (i) a clean, safe home with enough food, (ii) opportunities to learn, (iii) hobbies or intellectual pursuit, (iv) organized sport, (v) family routine or consistent rules [10] and vi) positive individual attributes [6, 14]. A recent scoping review of PCEs that focused on measurement and evidence highlighted the heterogeneity in the measurement of PCEs and emphasized the need for consistent operationalization of PCEs [17].

### Importance of Cumulative PCEs

If cumulative PCEs are proven effective in moderating the impact of ACEs using valid study designs, they could have a significant public health impact. Researchers can then explore whether enhancing non-family PCEs, such as the availability of a caring non-parent adult, positive community or neighborhood, school, and peer factors, could change the trajectory of children from disadvantaged backgrounds, such as those in out-of-home care or those exposed to high familial ACEs. This public health approach could potentially improve the resilience and well-being of large groups of young people at a time when mental health problems in youth are increasing.

### Research on PCEs

One of the earlier publications on PCEs used data from the ACEs study and showed that those with greater family strengths had reduced rates of risky sexual behaviors and teenage pregnancy [18]. Research evaluating individual domains of PCEs, such as parenting practices [19], school connectedness [20], non-parent adult [21] or positive neighborhood factors [22] indicated a significant association with better adolescent or adult health outcomes and psychosocial functioning. As PCE types (e.g., parenting, family, school, teacher, peer, neighborhood, and self-capacities and perception) tend to cluster together, studying one PCE type alone might lead to an overestimation of the impact [23].

Studies that used cumulative PCE scores also found a reduced risk of mental illness [8], cardiovascular disease [24], reconviction, and rearrest rates [25] but utilized retrospective reports of childhood experiences. A recent systematic review of 58 studies on the impact of PCEs on adult outcomes found that higher levels of PCEs were associated with lower odds for major depressive disorder, personality disorders, cigarette use, risky alcohol use, and general perceived stress. They also predicted better cardiovascular health, increased engagement in exercise, higher self-esteem, and better psychosocial functioning in adulthood [26].

Despite these important findings, this review had two major limitations [26]. First, all the included studies, except three, were cross-sectional and used retrospective measures to estimate PCEs. While retrospective reporting can allow an adult to recall their whole childhood experience, they are subject to recall bias and have a poor correlation with prospective reports of abuse and neglect [27]. Second, the review did not focus on the cumulative effects of individual PCEs. While the impact of one type of PCE could be small, the question of interest is whether the cumulative effect of PCEs could be substantial to both the individual and groups of individuals and offer opportunities for large-scale public health interventions.

Twenty-three studies in the systematic review by Han et al.[26] used statistical interactions and other analytical methods to evaluate the protective effects of PCEs among individuals exposed to childhood adversities. While only seven out of those twenty-three studies provided evidence for protective effects, such that the association between childhood adversity and adverse outcomes was lower in those exposed to high PCEs, seven others indicated worse outcomes with high PCEs, and the rest provided insignificant or mixed results. Despite the mixed findings, the authors concluded that PCEs are more likely to promote positive outcomes rather than moderate the effects of adversities [26].

In a scoping review on PCEs, 61 of the 66 publications measured PCEs retrospectively. Of the longitudinal studies, only one cohort study reported across three publications evaluated the effects of cumulative PCEs, and two other studies estimated the impact of individual PCEs. They noted that all included studies found evidence for promotive and/or protective effects of PCEs on behavioral and health outcomes [17].

### Gaps in the Literature and Aims of this Study

To summarise, previous reviews reported that cumulative PCEs appear to be associated with positive adolescent or adult outcomes. However, the existing literature is limited by largely cross-sectional studies or retrospective reports from longitudinal samples, and there are inherent challenges in interpreting the true impact of PCEs on long-term outcomes using such studies. There is a need to examine prospective reports of PCEs using longitudinal designs and ascertain which PCEs are likely to be the best predictors of long-term outcomes. Further, previous research on PCEs has predominantly focused on adults. However, adolescence is a critical period of development, with the peak age and median age of onset of mental disorders being 14.5 years and 18 years, respectively [28]. It is also a time when those affected by ACEs are likely to begin engaging in high-risk behaviors [29]. Studying longitudinal adolescent and adult cohorts is thus needed to better understand the protective impact of PCEs on long-term behaviors. Additionally, it is essential to examine studies that evaluate cumulative PCE scores, as an individual exposed to multiple ACEs may require multiple PCEs to cope effectively [30]. To address some of these gaps in the literature, this systematic review was conducted to test the following hypotheses using longitudinal studies that explored the cumulative impact of PCEs prospectively: (1) Cumulative PCEs would positively impact longitudinal mental, physical, and psychosocial outcomes. (2) Cumulative PCEs would moderate the impact of ACEs (3) Certain combinations of PCEs would predict better outcomes in longitudinal samples.

## Method

This systematic review adhered to Preferred Reporting in Systematic Reviews and Meta-Analysis (PRISMA) 2020 guidelines [31]. Please find the PRISMA checklist in Appendix 1**.**

### Eligibility Criteria

Studies that measured cumulative PCEs prospectively before age 18 and evaluated the influence of those scores on mental health, physical health, or psychosocial outcomes using a longitudinal design were included. Studies conducted in clinical samples, measured PCEs retrospectively, included only one type of PCE or conducted analysis based on individual PCE types rather than cumulative scores were excluded.

### Information Sources

A systematic search was conducted in the following databases: Pubmed, EMBASE, CINAHL, Cochrane, PsychINFO, and Scopus, and it was updated on 24 May 2024. Reference lists of eligible papers were screened for additional articles.

### Search Strategy

The search strategy was designed to find longitudinal studies that examined PCEs and their impact on mental and physical health or psychosocial outcomes. A search strategy was created for PubMed, using various terms for PCEs, child or teen, longitudinal or prospective study, and then modified for other databases. Some search terms were based on those used in previous publications known to the authors. Reference lists of eligible papers were screened for additional papers. The search strategies for each database are presented in Appendix 2**.**

### Selection Process

One author (KK) performed the initial screen (title & abstract) to shortlist articles for in-depth full-text review. Two authors (KK) and (SS) reviewed these articles independently, and decisions were based on consensus using the criteria outlined above. Disagreements were resolved through discussions with a senior author (JS).

### Data Extraction

One author (KK) extracted the data using a standardized data extraction sheet, as shown in Appendix 3, which was verified by another author (SS).

### Quality Assessment

The quality of each publication was rated independently using criteria based on the Joanna Briggs Institute (JBI) checklist for cohort studies (which is listed in Appendix 4) [32] by two authors (KK and SS) based on the following questions (i) Were the two groups recruited from the same population? (ii) Was exposure measured similarly in people assigned to exposed and unexposed groups? (iii) Were the exposures measured in a valid and reliable way? (iv) Were confounding factors identified? (v) Were strategies to deal with confounders stated? (vi) Were the groups free of the outcome at the start of the study or the moment of exposure? (vii) Were the outcome measures valid and reliable? (viii) Was the follow-up time reported and was it sufficient enough for outcomes to occur? (ix) Was the follow-up complete, and if not, were the reasons for the loss of follow-up described and explored? (x) Were strategies to address incomplete follow-up utilized? (xi) Was appropriate statistical analysis used? Decisions were based on consensus, and differences were resolved by further discussion with a senior author (JS).

### Synthesis

As the included papers measured different frameworks of PCEs and evaluated different outcomes using different measures across various age groups, it was not possible to synthesize the results in a meta-analysis.

The protocol for this systematic review was prospectively registered and can be accessed on PROSPERO (ID: CRD42022384775).

## Results

**Fig. 1:**
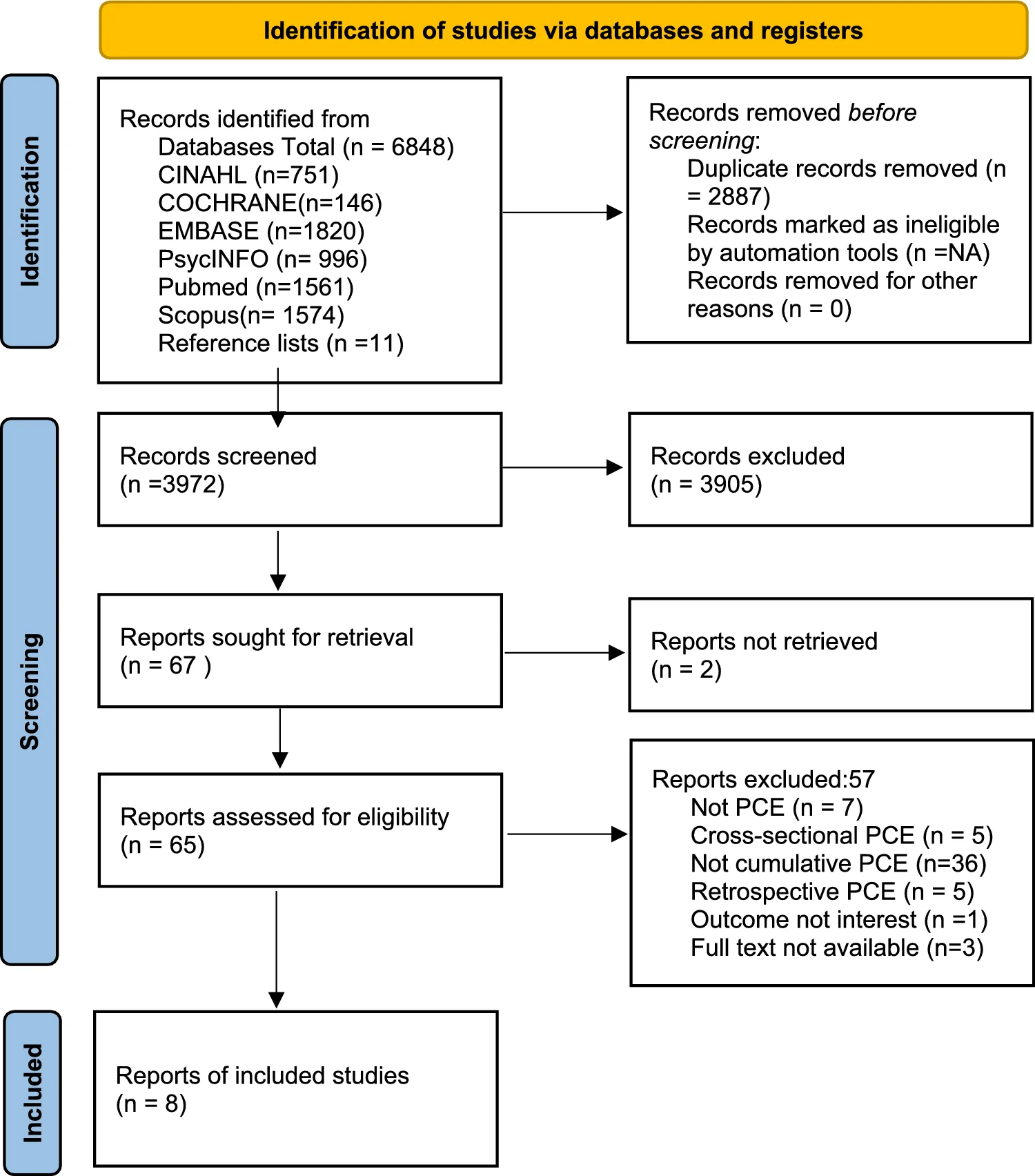
PRISMA Flow Chart

Following de-duplication, the search identified 3972 records. After screening titles and abstracts, 65 records were selected for in-depth review, and eight publications that used data from five cohorts were included. The Flourishing Families Project (FFP) was reported across three publications [33–35]. The five cohorts involved 16,451 participants. Figure 1 presents the PRISMA flow chart for the current review. Appendix 5 lists the excluded articles**.**

### Descriptive Information

#### Study Characteristics

Characteristics of the included studies are described in Table 2. Two of the four cohorts were from the United States [34, 36], and one each from Australia [15], Taiwan [37], and the United Kingdom [38]. One publication combined data from two cohorts conducted in the United Kingdom and Australia [39]. Sample sizes ranged from 489 [34] to 4875 [39].

**Table 2:** Study characteristics

Number	Author and year of publication	Cohort sample size	Setting sample country	Childhood adversities/ risk factors	Measures for adversities	Positive childhood experiences/ measures	Covariates/ mediators	Outcomes of interest/age	Findings
1	Crandall [33]^a^, Broadbent [34], Rollins [35]	489	Flourishing Families Project (Start 2007) waves 1–10 Adolescents with base age 10–13 Female 51% United States	ACEs	Adapted from ACEs scale. (Rated each wave 1–5)	Counter ACEs adapted from BCEs Scale (Exposure in the child measured by self and parent report) (Rated each wave 1–5)	Gender Age Race Sampling method Mediators Shame (SPS) Self-regulation (SRS)	5 Health indicators In wave 10 Depressive (CES-DC) Anxiety (SCAI) Body image (SATAQ) Substance use (ADUIS) Risky Sex (RSB) 10-year follow-up (age 20–23)	Counter-ACEs were predictive of lesser depression scores (β = − 0.11, p < .05), substance abuse (β = −0.17, p < 0.01), risky sex (β = −0.12, p < 0.05),, and a more positive body image (β = 0.15, p < 0.01) but no significant differences in anxiety scores (β = −0.10, p < 0.10) were noted. Counter ACEs associated with lower tobacco and alcohol use. Counter ACEs predicted shame negatively and positively predicted self-regulation. Shame mediated regulation between PCEs and depression. Self-regulation mediated relationship between ACEs, PCEs, and anxiety
2	Gondek [38]^b^	4490	ALSPAC birth cohort (Start 1991–1992) Children with base age 7 Female 51.3 United Kingdom	Parental Intimate Partner Violence	Exposure of IPV to parent based on self-report of violence (when the children were aged 2–9)	HOPE domains Positive Parenting Supportive relationships Supportive neighborhood Social engagement & enjoyment (Exposure in the child measured by self and parent report) (rated each wave ages 9–14)	Sex Family ethnicity Maternal partnership status Parent education Social class Financial difficulties Housing tenure Crowding index Smoking or alcohol use during pregnancy Mothers age at birth Parental mental health	Depression (SMFQ) 4–9 years follow up (age 18)	Each additional highly positive childhood experience (top quartile) was associated with −0.042 (95% CI: −0.060, −0.025) or 4.2% lower depression score
3	Guo [15]^c^	3111	LSAC birth cohort (Start 2004) Waves 1–8 Female 48.7% Australia			HOPE Domains Positive Parenting Supportive relationships Supportive neighborhood Social engagement & enjoyment) (Exposure in the child measured by self and parent report) (Rated each wave age 0–11)	Sex Ethnicity Socioeconomic status	Mental Health (SDQ) & Academic Skills (ARS-LL) follow up 3–14 years (age 14–15)	Higher cumulative PCEs at 10–11 years were associated with a medium to large reduction in mental health problems (β = −0.65 (95%CI: −0.81–0.50)) and a small reduction in impairment of academic skills (β = −0.02 (95% CI: −0.05, −0.00) at age 14–15)
4	Novak [36]	795	LONG SCAN (start 1991) Children exposed to maltreatment End age 16.29 Female 52% United States	ACEs	From CPS records and parent reports (ages 4–12)	Prosocial peer association Prosocial activities Positive parent relationship Neighborhood environment Importance to education Nonrelative adult mentor Self-report (age 12)	Family income Single parent home Gender Race Age	Self-reported delinquency and arrest follow-up 3- 4 years (age16)	Higher cumulative PCEs had significantly lower levels of delinquency (β = −0.31, p = .03) but not arrests (β = −0.07, p = 0.76)
5	Priest [39]^b,c^	1237 LSAC 3488 ALSPAC	LSAC birth cohort (start 2004). Waves 6 and 7 age (11–12) Female 51.1 Australia ALSPAC birth cohort (start 1991–92) Mean age 15.5 Base age 7 Female 51.3 United Kingdom	Parent separation, legal problems, violence, mental illness, substance abuse, harsh parenting, unsafe neighborhood, family death, and bullying	Rated each wave (ages 0–11) (LSAC) & (ages 0–15 (ALSPAC))	HOPE framework rated each wave Positive Parenting Supportive relationships Supportive neighborhood Social engagement & enjoyment (Ages 0–11 (LSAC) & 9–14 (ALSPAC))	Sex Socioeconomic index Maternal age at birth Ethnicity Birthweight Indoor smoking Child age at assessment	Inflammatory markers C-reactive protein(hsCRP) Glycoprotein acetyl (GlycA) (ages 11–12) follow up 1–11 years (LSAC) & (mean age 15.5, follow up 1–15 years (ALSPAC)	Cumulative PCE scores were associated with 22.1% lower hsCRP (95% CI: 49.0%, 4.7%) and 1.3% lower GlycA (95% CI: 2.7%, 0.2%), indicating lower levels of inflammation
6	Wu [37]	2841 Taiwan Youth Project (TYP)	TYP (Start 2000). Mean age 14 Taiwan			7 PCE items used in Bethell [8]	Age Gender Parental education Location Substance use Self-esteem Depressive symptoms Insomnia in adolescents	Persistent insomnia	Higher PCE scores were associated with lesser rates of persistent insomnia (Adjusted odds ratio = 0.93, 95% CI: 0.88, 0.98) [37]. While high PCEs mitigated the negative effects of ACEs, the impact of PCEs was inhibited in individuals with four or more ACEs

*ALSPAC* Avon Longitudinal Study of Parents and Children, *ARS-LL(subscale)* Academic Rating Scale of Language and Literacy, *CES-DC* Center for Epidemiological Studies Depression Scale for Children, *CPS* Child Protection System, *PCE* Positive Childhood Experiences, *HOPE* Health Outcomes From Positive Experiences, *LONGSCAN* Longitudinal Studies of Child Abuse and Neglect, *LSAC* Longitudinal Study of Australian Children, *RSB* Risky sexual behavior was based on items from the Three Cities Studies and National Survey of Family Growth, *SATAQ* Sociocultural Attitudes Towards Appearance Questionnaire, *SCAI* Spence Child Anxiety Inventory, *SDQ* Strengths & Difficulties Questionnaire, *SMFQ* Short Mood and Feelings Questionnaire

^a^Main publication; ^b^One of the papers linked to ALSPAC study; ^c^ One of the papers linked to LSAC study

#### Sample Characteristics

The sample size of studies ranged from 489 [34] to 4865 participants [39].The included cohorts had different types of samples. One cohort used a community sample from Washington State, United States [34], one included adolescents in Taiwan who were subsequently enrolled in college [37] and another used data from the Longitudinal Studies of Child Abuse and Neglect (LONGSCAN) that followed up on children referred to child protection services in North Carolina, United States [36]. The baseline age range of participants varied from 4–6 [36] to 13–15 years [37]. Two others, Longitudinal Study of Australian Children (LSAC) [15, 39] and Avon Longitudinal Study of Parents and Children (ALSPAC) [38] from the United Kingdom were birth cohorts.

#### PCE Types and Measures

Three publications included the Health Outcomes from Positive Experiences (HOPE framework [15, 38, 39], three publications used the Benevolent Childhood Experiences (BCEs) framework and measure [33–35], one used seven PCE items developed by Bethell et al. [8, 37] and another used a list of PCEs from the LONGSCAN study [36]. Different types of PCEs across studies are reported in Table 3.

**Table 3:** Types of positive childhood experiences (PCEs)

Study ID	Parent	Family routine/relationships	School	Teacher	Peer	Nonparent adult relationship	Positive self perception	Neighborhood
Crandall [34]	YES	YES	YES	YES	YES	NO	YES	NO
Gondek [38]	YES	YES	NO	YES	YES	NO	NO	YES
Guo [15]	YES	YES	NO	YES	YES	NO	NO	YES
Novak [36]	YES	NO	YES	NO	YES	YES	NO	YES
Priest [39]	YES	YES	NO	YES	YES	NO	NO	YES
Wu [37]	YES	YES	YES	NO	YES	YES	NO	NO

### Cumulative PCEs and Health Promotive Effects

The publications included in this review examined the relationship between PCEs and various outcomes across different contexts.

#### Mental Health Outcomes

Six of the eight included publications examined mental health outcomes. The mental health outcomes assessed were depression, anxiety, substance use, and global mental health. Two studies that evaluated the impact of cumulative PCEs on depression indicated promotive effects. While the cumulative counter-ACEs score predicted lesser depression scores (β =  −0.11, p < 0.05) after controlling for ACEs, in the FFP [34], each increase in PCE score among those exposed to parental intimate partner violence was associated with a 4.2% reduction in depression scores in adulthood in the ALSPAC birth cohort [38]. Broadbent et al.[33] found that PCEs were predictive of alcohol use (β = −0.13, p < 0.05) and tobacco use (β = −0.14, p < 0.05) [33]. Data from the LSAC study showed that PCE scores based on the HOPE framework were associated with medium to large reductions in general mental health problems as measured by the Strengths and Difficulties Questionnaire (SDQ) (β = −0.65, 95% CI: −0.81, −0.50). In contrast, counter-ACEs were not associated with reduced anxiety scores [34, 38]. One publication that explored the factors mediating the impact of PCEs on depression and anxiety found higher cumulative PCEs negatively correlated with shame ( r = −0.20, p < 0.001) and positively predicted self-regulation (r = 0.21, p < 0.001). While shame mediated the relationship between PCEs and depression (Z = −3.1, p < 0.01), self-regulation mediated the relationship between PCEs and anxiety (Z = −2.53, p < 0.05) [35].

#### Physical Health Outcomes

Two publications examined physical health outcomes. Based on data from two birth cohorts, cumulative PCEs were associated with reductions in high-sensitivity C-reactive protein (hs-CRP) (−22.1%, 95% CI: −49.0%, 4.7%) and glycoprotein acetyls (GlycA) (−1.3%, 95% CI: −2.7%, 0.2%) indicating less inflammation [39]. Higher PCE scores were also related to lesser rates of persistent insomnia (Adjusted odds ratio = 0.93, 95% CI: 0.88, 0.98) in emerging adulthood [37].

#### Psychosocial Outcomes

Three publications examined psychosocial outcomes. Cumulative PCEs were associated with a very small, non-significant reduction of impairment in academic skills in adolescents from the general population( β = −0.02, 95% CI: −0.05, 0.00) [15]. Among teens who were known to the juvenile justice system and had high exposure to abuse and family dysfunction, those with higher PCEs reported significantly reduced levels of delinquency (β = −0.31, p = 0.03) but not arrests (β = −0.07, p = 0.76) [36]. Finally, one article found small associations between cumulative PCE scores and reduced risky sexual behaviors (β = −0.12, p < 0.05) and positive body image in adulthood (β = 0.15, p < 0.01) [34].

#### Adverse Childhood Experiences

ACEs were common in all five cohorts included in this review, but there were some variabilities in the types of ACEs reported by the studies. Children who experienced maltreatment or were at risk of maltreatment were included in the LONGSCAN study. While 37% experienced more than four ACEs, 58% experienced more than two ACEs. In this study, 33% had alleged physical or emotional abuse, while 15% had alleged sexual abuse [36]. While, among participants of the LSAC study, family death (27.4%) was the commonest ACE,family mental illness (42.8%) was the most common ACE in those included in the ALSPAC study. Over half of the children experienced more than two ACEs in both LSAC (50.3%) and ALSPAC (55.1%) studies. Details about childhood sexual abuse were not collected in LSAC. Though nearly 4% of participants reported childhood sexual abuse in ALSPAC [40], only physical abuse (9.3%) was included in the analysis [39]. In the FFP, participants experienced a cumulative average of 2.7 ACEs by wave 5 (age range 15–18). Family mental illness (51%) was the commonest ACE, followed by physical abuse (49%) and emotional abuse (40%), but respondents were not asked about sexual abuse [34]. On average, the Taiwan Youth Project participants had an ACE score of 1.26, and 7.29% experienced four or more ACEs [37].

#### Interactive Effects of PCEs (Moderation)

Four publications evaluated the moderating effects of PCEs on those exposed to adversities. [36–39]. Gondek [38] estimated the coefficients quantifying the strength of the association between PCEs and depressive symptoms in those exposed to IPV. They found that each additional PCE was associated with 3.8% lower depression scores in that group. Novak [36] divided their sample into two subgroups: those with two or fewer ACEs and those with three or four ACEs. While four or more PCEs predicted reduced rates for self-reported delinquency in both groups, higher PCEs did not moderate rearrest rates. Priest [39] analyzed the effect of PCEs by studying the impact on subgroups stratified by the number of ACEs. They found that the reduction in inflammation linked to PCEs was more pronounced among those with fewer concurrent ACEs. Wu [37] stratified their sample into those with PCE scores above three and others. Among those in the group with three or more PCEs, ACEs showed no significant relationship with persistent insomnia. Crandall [34] studied the interaction effects between ACEs and PCEs. They calculated the difference score between PCEs and ACEs and then found that as the difference score increased, participants experienced better mental health and psychosocial outcomes.

### Quality of Included Studies

The quality of the included articles was rated against JBI criteria for cohort studies and is shown in Table 4 [32]. All included articles had both exposed and non-exposed (to PCEs) participants within the same group, accounted for confounders, and were free of outcomes when the exposure (to PCEs) started. Three publications adapted items from the validated BCEs scale to develop the PCE measure (Counter-ACEs) but did not evaluate the reliability and validity [33–35]. Three papers used the PCE measure developed from the HOPE framework and used Confirmatory Factor Analysis (CFA) and found a good fit for the underlying factor structure of the model [15, 38, 39]. One paper estimated intra-class correlation for each domain of PCEs but did not test for construct validity [36]. Wu [41] employed seven PCE items developed by Bethell et al., who verified intra-class correlation and completed Principal Component Analysis (PCA) of those items [8]. All PCE measures had good predictive validity.

**Table 4:** Quality rating of included studies^a^

Study	Exposed & non exposed from similar groups	Exposure (PCE) measured similarly	Exposure (PCE) measure		Confounding identified	Confounding dealt with	Free of outcome at start	Outcome measure		Follow up time Adolescents/ adults	Lost to follow		Adjusted for dropouts	Appropriate statistical tests	Rating Max 14
			Reliable	Valid				Reliable	Valid		< 20%	Impact explored			
Crandall [34]^b^	Yes	Yes	No	No	Yes	Yes	Yes	Yes	Yes	10 years Adults	Yes	NA	NA	Yes	12^d^
Gondek [38]	Yes	Yes	Yes	Yes	Yes	Yes	Yes	Yes	Yes	4–9 years Adults ^c^	No	Yes	No	Yes	12
Guo [15]	Yes	Yes	Yes	Yes	Yes	Yes	Yes	Yes	Yes	3–13 years Adolescents	No	No	No	Yes	10
Novak [36]	Yes	Yes	Yes	No	Yes	Yes	Yes	No	No	3–4 years Adolescents	NA	NA	NA	Yes	10^d^
Priest [39]	Yes	Yes	Yes	Yes	Yes	Yes	Yes	Yes	Yes	1–14 years Adolescents	No	No	No	Yes	10
Wu [37]	Yes	Yes	Yes	Yes	Yes	Yes	Yes	No	No	7–8 years Adults	Yes	NA	NA	Yes	12^d^

^a^Joanna Briggs Institute quality rating for cohort studies; ^b^ Same rating for Broadbent [33] and Rollins [35] (al from same sample); ^c^ outcomes at 18 years of age; ^d^ NA counted as 1

While six publications utilized valid and reliable outcome measures [15, 33–35, 38, 39], two used self-reporting for outcomes without testing for reliability or validity [36, 41]. The duration of follow-up varied widely from four [36] to fourteen years [15, 39] and attrition ranged from 7.4% [34] to 70% [38]. For the biological measures of health, only about 24% of participants in LSAC and ALSPAC had blood samples taken [39].

## Discussion

### PCEs and Health Outcomes

In this systematic review, cumulative PCEs in childhood were associated longitudinally with modest benefits to mental health and psychosocial outcomes in adolescence and young adulthood (ages 15 to 23 years). We found two publications on physical health outcomes that suggested that PCEs were correlated with a lower risk of persistent insomnia and lower levels of inflammation. A previous systematic review that predominantly included cross-sectional and retrospective studies also showed the beneficial effects of PCEs on physical health, mental health, and psychosocial functioning in adults [26].

### Protective Effects of PCEs

In the current review, four studies that evaluated the moderating effects of cumulative PCEs and one that studied interaction effects yielded positive results. These findings support our second hypothesis that cumulative PCEs may reduce poor mental health and psychosocial outcomes associated with adversities in childhood. This is congruous with previous research suggesting that ACEs are not necessarily deterministic of poor health outcomes [42]. Similarly, two studies that measured PCEs prospectively found that PCEs moderated the impact of childhood adversities [25, 43] but one study that used retrospective measures of PCEs indicated no protective effects [44].

When Han and colleagues [26] evaluated interactions between childhood adversities and PCEs., only seven out of twenty-three studies showed evidence of significant interaction. This could be due to the inclusion of studies that did not evaluate cumulative PCE scores, as an individual exposed to multiple ACEs may require multiple PCEs to cope effectively [30]. Using retrospective measures that were subject to recall bias could be another reason.

Further, the results from three included studies [34, 37, 39] indicated that PCEs may moderate the effects better in those exposed to less severe adversities. These findings are consistent with two retrospective studies that evaluated the impact of cumulative PCEs on various health diagnoses and obesity [45, 46] and a study where the greatest caregiver warmth protected cardiovascular health in those with the lowest child abuse exposure and not in those with the most severe child abuse exposure [47].

### PCE Types

The papers in our review used four different PCE frameworks: a modified HOPE framework, a BCEs framework, and a PCE list from Bethell et al. [8], and the PCEs list that was curated for the LONGSCAN data set. Five articles only used items that were part of safe, stable nurturing relationships and environment frameworks, including various social engagements but not positive self-qualities or stable family routines. Interestingly, Guo et al. [15] deliberately excluded the “social and emotional competencies” while validating the HOPE framework, arguing that those could be outcomes secondary to PCEs. Only the study by Crandall et al. [34] the BCE framework also included measures on positive self-qualities and stable family routines, but their results were in line with others. These results indicate that safe, stable, nurturing relationships may form the core component of the PCE construct. This partly verifies our third hypothesis that certain combinations of PCEs would predict better outcomes in longitudinal samples.

### Quality of Studies

Overall, the included studies were of good quality as rated by the criteria of JBI quality rating for cohort studies, but there were some deficits [32]. Two studies did not use valid measures for PCEs [34, 36] and two studies did not use valid or reliable outcome measures for outcomes [36, 37]. As expected, some of these longitudinal studies had large attrition, although research suggests this does not necessarily lead to bias [48].

### Future Research

The research into cumulative PCEs is in its early stages, and some gaps require further study. With the assumption that each type of PCE is equally important for a specific outcome, the potential for differential influence of each PCE type is ignored. It is unclear if non-family PCEs (school, neighborhood, peers, non-parent adult, and positive self-perception) are effective in those without PCEs within their families. There are also uncertainties on how to measure PCEs. One study counted individual PCE domains as present if the participants scored more than the average of the sum for all the items in that domain, even in one wave of data collection [34]. Others had cut-offs dichotomized for each domain at the 75th percentile of summed scores [15, 36] and estimated associations separately for each wave of data collection and in combination [15]. Both approaches may have captured those exposed to higher-intensity PCEs. This highlights the possibility that the mere presence of PCEs may not influence outcomes but depend on the greater quality and intensity of those experiences.

Given the high worldwide prevalence of mental disorders, there is strong interest in translating learnings from research on PCEs into meaningful public health interventions [49]. Healthy Steps, The Strengthening Families Approach and Protective Factors Framework ™, the HOPE framework, the Families and Schools Together program (FAST), Restorative Circles, and Circle of Courage are some of the initiatives and interventions that have been recommended to promote PCEs, to mitigate the impact of ACEs and thereby disrupt the link between adversities and negative stress-related outcomes [50–55]. This has led to predictions that the promotion of PCEs might improve mental and physical health at a population level [56].

### Limitations

While we were able to investigate cumulative PCEs from longitudinal studies in the current review, there are some limitations. Only eight publications from five longitudinal datasets were available for inclusion, and further research using prospective longitudinal data is required. Inherent to long-term prospective studies, some had high attrition rates. Most studies evaluated outcomes in adolescence but were of high quality. Finally, they were all from developed countries limiting generalizability to other populations.

### Summary

This systematic review of longitudinal studies that evaluated prospectively measured PCEs strengthens the previous findings from cross-sectional and retrospective studies that PCEs may be associated with long-term positive mental health, physical health, and psychosocial outcomes. In contrast to previous research, this review also highlights the potential protective effects of PCEs against various adverse experiences. Most included studies had high-quality ratings based on the JBI rating for cohort studies, offering us confidence in the results. There is a lack of consensus on what components constitute PCEs. Interestingly, all studies except one included only the safe, stable, nurturing relational component of PCEs, indicating its central role in PCEs. Most studies in this review captured higher exposure to PCEs, suggesting that the mere presence of PCEs may not be adequate to offer long-term benefits, and the results would depend on the quality of those experiences. PCEs could be integrated into public health interventions to protect against the negative effects of adverse childhood experiences and promote overall well-being. However, further research is required to replicate the existing findings, clarify the key components of PCEs, resolve the issues with the measurement of PCEs, and improve the generalizability and validity of PCEs by conducting studies globally, including in developing countries.

## Supplementary Information

Below is the link to the electronic supplementary material.

Supplementary file1 (DOCX 32 KB)

Supplementary file2 (DOCX 23 KB)

Supplementary file3 (DOCX 13 KB)

Supplementary file4 (PDF 720 KB)

Supplementary file5 (DOCX 28 KB)

## Data Availability

No datasets were generated or analysed during the current study.
