# Supplementary material for: Impact of Positive Childhood Experiences (PCEs): A Systematic Review of Longitudinal Studies
Source: Child Psychiatry Hum Dev. 2025 Jan 6;57(5):1565–80. doi: 10.1007/s10578-024-01807-x (PMC13615033; doi:10.1007/s10578-024-01807-x)
Supplement: Supplementary file 3 [file 10578_2024_1807_MOESM2_ESM.docx]

Appendix I : Search Strategy

PubMed:

positive childhood experience*" OR "advantageous childhood experience*" OR "counter ace*" OR "benevolent childhood experience*" OR "Positive parenting" OR "Supportive relationship*" OR "Supportive neighborhood*" OR "Social engagement" OR "Family cohesion" OR "parental affection" OR "positive parental involvement" OR connectedness OR "protective and compensatory experience*" OR "positive caregiving experience*"[All Fields] OR "family support"[All Fields] OR "nurtur*"[All Fields]

AND

child*[Title/Abstract] OR boy[Title/Abstract] OR boys[Title/Abstract] OR girl*[Title/Abstract] OR "teen*"[Title/Abstract]

AND

(longitudinal study) OR "cross lagged study" [All Fields] OR "National Survey of Midlife Development"[All Fields] OR (waves interview*) OR "National Social Life, Health, and Aging Project"[All Fields] OR "Midlife and Aging in the United States study"[All Fields] OR (the Flourishing Families Project) OR "prospective studies"[MeSH Terms] OR "prospective studies"[All Fields] OR "prospective study"[All Fields] OR "cohort studies"[MeSH Terms] OR "cohort studies"[All Fields] OR "cohort study"[All Fields] OR "cohort analysis"[All Fields] OR "follow up studies"[MeSH Terms] OR "follow up studies"[All Fields] OR "follow up study"[All Fields]

1156

EMBASE

Embase Session Results

No.

Query

Results

1,507

#36

#23 AND #35

3,427,382

#35

#24 OR #25 OR #26 OR #27 OR #28 OR #29 OR #30 OR #31 OR #32 OR #33 OR #34

1,962,403

#34

'follow up study'/exp OR 'follow up study'

1,956,190

#33

'follow up studies'/exp OR 'follow up studies'

992,269

#32

'cohort study'/exp OR 'cohort study'

928,616

#31

'cohort analysis'/exp OR 'cohort analysis'

948,618

#30

'cohort studies'/exp OR 'cohort studies'

1

#29

'midlife and aging in the united states study'

850,480

#28

'prospective studies'/exp OR 'prospective studies'

878,421

#27

'prospective study'/exp OR 'prospective study'

150

#26

'national survey of midlife development'

44

#25

'cross lagged study'

205,373

#24

'longitudinal study'/exp OR 'longitudinal study'

9,355

#23

#17 AND #22

2,317,907

#22

#18 OR #19 OR #20 OR #21

47,424

#21

teen*:ti,ab

227,061

#20

girl*:ti,ab

233,101

#19

boy*:ti,ab

2,095,170

#18

child*:ti,ab

33,423

#17

#1 OR #2 OR #3 OR #4 OR #5 OR #6 OR #7 OR #8 OR #9 OR #10 OR #11 OR #12 OR #13 OR #14 OR #15 OR #16

10,410

#16

nurtur*

10,077

#15

'family support'

43

#14

'positive caregiving experience*'

2

#13

'protective and compensatory experience*'

6,761

#12

connectedness

9

#11

'positive parental involvement'

53

#10

'parental affection'

1,333

#9

'family cohesion'

2,532

#8

'social engagement'

34

#7

'supportive neighborhood*'

1,110

#6

'supportive relationship*'

1,486

#5

'positive parenting'

15

#4

'benevolent childhood experience*'

36

#3

'counter ace*'

4

#2

'advantageous childhood experience*'

64

#1

'positive childhood experience*'

Cochrane

**Date Run:	02/12/2022 04:53:34**

**Comment:**

**ID	Search	Hits**

**#1	"positive childhood experience*" OR "advantageous childhood experience*" OR "counter ace*" OR "benevolent childhood experience*" OR "Positive parenting" OR "Supportive relationship*" OR "Supportive neighborhood*" OR "Social engagement" OR "Family cohesion" OR "parental affection" OR "positive parental involvement" OR connectedness OR "protective and compensatory experience*" OR "positive caregiving experience*" OR "family support" OR nurtur*	2727**

**#2	(child* OR boy OR boys OR girl* OR teen*):ab (Word variations have been searched)	136583**

**#3	(child* OR boy OR boys OR girl* OR teen*):ti (Word variations have been searched)	82737**

**#4	#2 OR #3	156403**

**#5	#1 AND #4	1135**

**#6	MeSH descriptor: [Longitudinal Studies] explode all trees	6606**

**#7	MeSH descriptor: [Prospective Studies] explode all trees	100602**

**#8	MeSH descriptor: [Cohort Studies] explode all trees	161812**

**#9	MeSH descriptor: [Follow-Up Studies] explode all trees	62270**

**#10	"longitudinal study" OR “cross lagged study” OR "National Survey of Midlife Development" OR (waves interview*) OR "National Social Life, Health, and Aging Project" OR "Midlife and Aging in the United States study" OR "prospective studies" OR "prospective study" OR "cohort studies" OR "cohort study" OR "cohort analysis" OR "follow up studies" OR "follow up study"	253775**

**#11	{OR #6-#10}	262273**

**#12	#5 AND #11	133**

**Scopus**

(((TITLE(child* OR boy OR boys OR girl* OR teen*)) OR (ABS(child* OR boy OR boys OR girl* OR teen*))) AND (TITLE-ABS-KEY("positive childhood experience*" OR "advantageous childhood experience*" OR "counter ace*" OR "benevolent childhood experience*" OR "Positive parenting" OR "Supportive relationship*" OR "Supportive neighborhood*" OR "Social engagement" OR "Family cohesion" OR "parental affection" OR "positive parental involvement" OR connectedness OR "protective and compensatory experience*" OR "positive caregiving experience*" OR "family support" OR nurtur*))) AND (TITLE-ABS-KEY("longitudinal study" OR "cross lagged study" OR "National Survey of Midlife Development" OR (waves interview*) OR "National Social Life, Health, and Aging Project" OR "Midlife and Aging in the United States study" OR "prospective studies" OR "prospective study" OR "cohort studies" OR "cohort study" OR "cohort analysis" OR "follow up studies" OR "follow up study"))

CiNAHL

| S5 | S3 AND S4 | Expanders - Apply equivalent subjects 663 Search modes - Boolean/Phrase | Interface - EBSCOhost Research Databases Search Screen - Advanced Search Database - CINAHL Complete | 663 |
| --- | --- | --- | --- | --- |
| S4 | "longitudinal study" OR “cross lagged study” OR "National Survey of Midlife Development" OR (waves interview*) OR "National Social Life, Health, and Aging Project" OR "Midlife and Aging in the United States study" OR "prospective studies" OR "prospective study" OR "cohort studies" OR "cohort study" OR "cohort analysis" OR "follow up studies" OR "follow up study" | Expanders - Apply equivalent subjects 591,243 Search modes - Boolean/Phrase | Interface - EBSCOhost Research Databases Search Screen - Advanced Search Database - CINAHL Complete | 591,243 |
| S3 | S1 AND S2 | Expanders - Apply equivalent subjects 5,744 Search modes - Boolean/Phrase | Interface - EBSCOhost Research Databases Search Screen - Advanced Search Database - CINAHL Complete | 5,744 |
| S2 | TI ( child* OR boy OR boys OR girl* OR "teen* ) OR AB ( child* OR boy OR boys OR girl* OR "teen* ) OR AB ( child* OR boy OR boys OR girl* OR "teen* ) | Expanders - Apply equivalent subjects 622,631 Search modes - Boolean/Phrase | Interface - EBSCOhost Research Databases Search Screen - Advanced Search Database - CINAHL Complete | 622,631 |
| S1 | "positive childhood experience*" OR "advantageous childhood experience*" OR "counter ace*" OR "benevolent childhood experience*" OR "Positive parenting" OR "Supportive relationship*" OR "Supportive neighborhood*" OR "Social engagement" OR "Family cohesion" OR "parental affection" OR "positive parental involvement" OR connectedness OR "protective and compensatory experience*" OR "positive caregiving experience*" OR "family support" OR nurtur* | Expanders - Apply equivalent subjects 20,307 Search modes - Boolean/Phrase | Interface - EBSCOhost Research Databases Search Screen - Basic Search Database - CINAHL Complete | 20,307 |

PsycInfo

| S5 | S3 AND S4 | Expanders - Apply equivalent subjects 895 Search modes - Boolean/Phrase | Interface - EBSCOhost Research Databases Search Screen - Advanced Search Database - CINAHL Complete | 663 |
| --- | --- | --- | --- | --- |
| S4 | "longitudinal study" OR “cross lagged study” OR "National Survey of Midlife Development" OR (waves interview*) OR "National Social Life, Health, and Aging Project" OR "Midlife and Aging in the United States study" OR "prospective studies" OR "prospective study" OR "cohort studies" OR "cohort study" OR "cohort analysis" OR "follow up studies" OR "follow up study" | Expanders - Apply equivalent subjects 177,855 Search modes - Boolean/Phrase | Interface - EBSCOhost Research Databases Search Screen - Advanced Search Database - CINAHL Complete | 591,243 |
| S3 | S1 AND S2 | Expanders - Apply equivalent subjects 15,370 Search modes - Boolean/Phrase | Interface - EBSCOhost Research Databases Search Screen - Advanced Search Database - CINAHL Complete | 5,744 |
| S2 | TI ( child* OR boy OR boys OR girl* OR "teen* ) OR AB ( child* OR boy OR boys OR girl* OR "teen* ) OR AB ( child* OR boy OR boys OR girl* OR "teen* ) | Expanders - Apply equivalent subjects 821,440 Search modes - Boolean/Phrase | Interface - EBSCOhost Research Databases Search Screen - Advanced Search Database - CINAHL Complete | 622,631 |
| S1 | "positive childhood experience*" OR "advantageous childhood experience*" OR "counter ace*" OR "benevolent childhood experience*" OR "Positive parenting" OR "Supportive relationship*" OR "Supportive neighborhood*" OR "Social engagement" OR "Family cohesion" OR "parental affection" OR "positive parental involvement" OR connectedness OR "protective and compensatory experience*" OR "positive caregiving experience*" OR "family support" OR nurtur* | Expanders - Apply equivalent subjects 41,886 Search modes - Boolean/Phrase | Interface - EBSCOhost Research Databases Search Screen - Basic Search Database - CINAHL Complete | 20,307 |
