# Supplementary material for: Impact of Positive Childhood Experiences (PCEs): A Systematic Review of Longitudinal Studies
Source: Child Psychiatry Hum Dev. 2025 Jan 6;57(5):1565–80. doi: 10.1007/s10578-024-01807-x (PMC13615033; doi:10.1007/s10578-024-01807-x)
Supplement: Supplementary file 4 [file 10578_2024_1807_MOESM3_ESM.docx]

**Appendix 2: Data Extraction**

**Study ID	Year**

**Number:**

**Setting:**

**Population ( Age, Gender):**

**Country:**

**Childhood Adversities OR Risk factors:**

**Measure for Childhood adversities:**

**Positive Childhood Experiences measure:**

**Covariates / Mediators:**

**Outcomes of interest (age/grade):**

**Findings:**
