# Supplementary material for: Impact of Positive Childhood Experiences (PCEs): A Systematic Review of Longitudinal Studies
Source: Child Psychiatry Hum Dev. 2025 Jan 6;57(5):1565–80. doi: 10.1007/s10578-024-01807-x (PMC13615033; doi:10.1007/s10578-024-01807-x)
Supplement: Supplementary file 5 [file 10578_2024_1807_MOESM5_ESM.docx]

| SNo | Study | Reason |
| --- | --- | --- |
| 1 | Atkinson 2023 | Not PCE |
| 2 | Babinski 1995 | Not cumulative PCE |
| 3 | Bacio 2010 | Not PCE |
| 4 | Baglivio 2021 | Follow up less than 12 months cross-sectional |
| 5 | Boehm 2022 | Not PCE conference proceeding full text not available |
| 6 | Calvin 2015 | Not PCE |
| 7 | Chang 2022 | Linked Wang 2021 Not cumulative PCE |
| 8 | Chen 2019 | Not cumulative PCE |
| 9 | Chiang 2022 | Not cumulative PCE |
| 10 | Conger 2002 | Not cumulative PCE |
| 11 | Coronado 2024 | Cross-sectional |
| 12 | Craig 2022 | Follow up less than 12 months cross sectional |
| 13 | Dansby Olufowote 2020 | Not cumulative PCE |
| 14 | Davies 2017 | Not cumulative PCE |
| 15 | Davis 2019 | Not cumulative PCE |
| 16 | Davis 2023 | No cumulative PCE |
| 17 | De Luca 2021 | Not cumulative PCE |
| 18 | Ehrlich 2015 | Not cumulative PCE |
| 19 | Geng 2021 | Cross-sectional |
| 20 | Gutierrez 2022 | Not cumulative PCE |
| 21 | Hadi 2022 | Same as Dansby Olufowote 2020 Not cumulative PCE |
| 22 | Herman 2024 | Retrospective |
| 23 | Hietanen 2016 | Not cumulative PCE |
| 24 | Hoyt 2012 | Not cumulative PCE |
| 25 | Hu 2023 | Not PCE |
| 26 | Huang 2023 | Retrospective |
| 27 | Huibregtse 2012 | Not PCE |
| 28 | Hunt 2023 | Not cumulative PCE |
| 29 | Jester 2014 | Not cumulative PCE |
| 30 | Jones 2019 | Full text not available |
| 31 | Kaman 2021 | Not cumulative PCE |
| 32 | Katainen 1999 | Not cumulative PCE |
| 33 | Koshy 2021 | Not PCE |
| 34 | Kosterman 2011 | Not cumulative PCE |
| 35 | La Charite 2024 | Not cumulative PCE |
| 36 | Lee 2015 | Not cumulative PCE |
| 37 | Lee 2021 | Not cumulative PCE |
| 38 | Lyu 2017 | Not cumulative PCE |
| 39 | Mahatmya2012 | Not outcome |
| 40 | Mason 2022 | Retrospective |
| 41 | Meinck 2021 | Not cumulative PCE |
| 42 | Orbuch 2021 | Not cumulative PCE |
| 43 | Reynolds 2011 | Not PCE |
| 44 | Skodol 2007 | Retrospective |
| 45 | Slopen 2017 | Retrospective |
| 46 | Sonuga-barke 2017 | Not cumulative PCE |
| 47 | Thomann 2014 | Not cumulative PCE |
| 48 | Topitzes 2010 | Not cumulative PCE |
| 49 | Treios-Castillo 2011 | Unable to get full text. |
| 50 | Triolo 2019 | Not cumulative PCE |
| 51 | Triolo 2020 | Not cumulative PCE |
| 52 | Van Delft 2016 | Not cumulative PCE |
| 53 | Van Harmelen 2017 | Not cumulative PCE |
| 54 | Wang 2021 | Not cumulative PCE |
| 55 | Williams 2015 | Not cumulative PCE |
| 56 | Zhang 2021 | Cross sectional |
| 57 | Zhu 2023 | Not cumulative PCE |

References:

Atkinson, L., Joshi, D., Raina, P., Griffith, L. E., Macmillan, H., & Gonzalez, A. (2021). Social engagement and allostatic load mediate between adverse childhood experiences and multimorbidity in mid to late adulthood: The Canadian Longitudinal Study on Aging [Article]. *Psychological Medicine*. https://doi.org/10.1017/S0033291721003019

Babinski, L. M. (1995). *Childhood adhd and aggression as predictors of later criminal activity: A longitudinal study of risk and protective factors from early childhood through young adulthood* ProQuest Information & Learning]. psyh. https://search.ebscohost.com/login.aspx?direct=true&db=psyh&AN=1995-95002-092&site=ehost-live

Bacio, G. A., Mays, V. M., & Lau, A. S. (2010). Drinking initiation and problematic alcohol use among Latino adolescents of distinct generations: Examining explanations for the immigrant paradox [Conference Abstract]. *Alcoholism: Clinical and Experimental Research, 34*(6), 120A. https://doi.org/10.1111/j.1530-0277.2010.01210.x

Baglivio, M. T., & Wolff, K. T. (2021). Positive Childhood Experiences (PCE): Cumulative Resiliency in the Face of Adverse Childhood Experiences. *Youth violence and juvenile justice, 19*(2), 139-162. https://doi.org/10.1177/1541204020972487

Boehm, J. K., Sumner, J. A., Qureshi, F., Chen, R., & Suglia, S. (2020). Hardships and Assets in Childhood: Associations with Health Outcomes Across the Lifespan [Conference Abstract]. *Psychosomatic Medicine, 82*(6), A157-A158. https://doi.org/10.1097/PSY.0000000000000835

Calvin, C. M., Batty, D., Brett, C. E., Deary, I. J., & Batty, G. D. (2015). Childhood Club Participation and All-Cause Mortality in Adulthood: A 65-Year Follow-Up Study of a Population-Representative Sample in Scotland. *Psychosomatic Medicine, 77*(7), 712-720. https://doi.org/10.1097/PSY.0000000000000210

Chang, Y., Yoon, S., Maguire-Jack, K., & Lee, J. (2022, Dec 20). Family-, School-, and Neighborhood-Level Predictors of Resilience for Adolescents with a History of Maltreatment. *Children (Basel), 10*(1). https://doi.org/10.3390/children10010001

Chen, P., & Harris, K. M. (2019, Dec 1). Association of Positive Family Relationships With Mental Health Trajectories From Adolescence to Midlife. *JAMA Pediatr, 173*(12), e193336. https://doi.org/10.1001/jamapediatrics.2019.3336

Chiang, S. C., & Bai, S. (2022, Apr). Internalizing symptoms among Taiwanese adolescents in poverty: Longitudinal influences of neighborhood, family, and school. *J Adolesc, 94*(3), 390-400. https://doi.org/10.1002/jad.12030

Conger, R. D., & Conger, K. J. (2002). Resilience in Midwestern families: Selected findings from the first decade of a prospective, longitudinal study. *Journal of Marriage and Family, 64*(2), 361-373.

Coronado, H., Bonilla, G. S., Shircliff, K., Sims, I., Flood, E., Cooley, J. L., & Cummings, C. (2024, May 7). Considering the associations of adverse and positive childhood experiences with health behaviors and outcomes among emerging adults. *J Exp Child Psychol, 244*, 105932. https://doi.org/10.1016/j.jecp.2024.105932

Craig, J. M., Wolff, K. T., & Baglivio, M. T. (2022). Clustering of adverse and positive childhood experiences: The nature and correlates of risk and protective factors [Article]. *Child Abuse and Neglect, 134*. https://doi.org/10.1016/j.chiabu.2022.105878

Dansby Olufowote, R. A., Soloski, K. L., Gonzalez-Casteñeda, N., & Hayes, N. D. (2020). An accelerated latent class growth curve analysis of adolescent bonds and trajectories of depressive symptoms. *Journal of Child and Family Studies, 29*, 292-306.

Davies, D., Consortium, N., Fletcher, P., Goodyer, I., & Jones, P. (2017). Different longitudinal relationships between childhood adversity, adolescent/adult social support, and dimensions of depressive and psychotic symptoms in 2 general population cohorts [Conference Abstract]. *Schizophrenia Bulletin, 43*, S115. https://www.embase.com/search/results?subaction=viewrecord&id=L616524979&from=export

Davis, A. L., & McQuillin, S. D. (2023). Exploring changes in the teacher-child relationship and children's educational expectations [Article]. *Journal of Applied Developmental Psychology, 87*, Article 101564. https://doi.org/10.1016/j.appdev.2023.101564

Davis, J. P., Ports, K. A., Basile, K. C., Espelage, D. L., & David-Ferdon, C. F. (2019, Dec). Understanding the Buffering Effects of Protective Factors on the Relationship between Adverse Childhood Experiences and Teen Dating Violence Perpetration. *J Youth Adolesc, 48*(12), 2343-2359. https://doi.org/10.1007/s10964-019-01028-9

De Luca, S. M., Caramanis, C., & Zhang, A. (2021). A longitudinal study examining the associations of bullying victimization and suicidal ideation among sexual minority adolescents. *Suicide and Life‐Threatening Behavior, 51*(6), 1138-1147.

Ehrlich, K. B., Sumner, J. A., Hoyt, L. T., McDade, T. W., & Adam, E. K. (2015). Quality of relationships with parents and friends in adolescence predicts metabolic risk in young adulthood [Article]. *Health Psychology, 34*(9), 896-904. https://doi.org/10.1037/hea0000213

Geng, F., Zou, J., Liang, Y., Zhan, N., Li, S., & Wang, J. (2021). Associations of positive and adverse childhood experiences and adulthood insomnia in a community sample of Chinese adults [Article]. *Sleep Medicine, 80*, 46-51. https://doi.org/10.1016/j.sleep.2021.01.022

Gutierrez Eisman, G. M. (2022). *Promotive and risk factors associated with adolescent alcohol and cigarette initiation: A longitudinal analysis* ProQuest Information & Learning]. psyh. https://search.ebscohost.com/login.aspx?direct=true&AuthType=shib&db=psyh&AN=2022-56509-051&site=ehost-live&custid=s1097571

Hadi, N., Zaru, R., Trejos-Castillo, E., & Rosales, O. (2022). Children of the incarcerated: The role of parenting, school, and social support in Caucasian and African American youth outcomes. In *Advances in Sociology Research. Volume 39* (pp. 133-158). Nova Science Publishers, Inc.

Herman, K. A., Hautala, D. S., Aulandez, K. M. W., & Walls, M. L. (2024, Feb 29). The resounding influence of benevolent childhood experiences. *Transcult Psychiatry*, 13634615231192006. https://doi.org/10.1177/13634615231192006

Hietanen, H., Aartsen, M., Kiuru, N., Lyyra, T.-M., & Read, S. (2016). Social engagement from childhood to middle age and the effect of childhood socio-economic status on middle age social engagement: results from the National Child Development study. *Ageing & Society, 36*(3), 482-507. https://doi.org/10.1017/S0144686X1400124X

Hoyt, L. T., Chase-Lansdale, P. L., McDade, T. W., & Adam, E. K. (2012, Jan). Positive youth, healthy adults: does positive well-being in adolescence predict better perceived health and fewer risky health behaviors in young adulthood? *J Adolesc Health, 50*(1), 66-73. https://doi.org/10.1016/j.jadohealth.2011.05.002

Hu, N., Gelaw, Y. A., Katz, I., Fernandez, E., Falster, K., Hanly, M., Newton, B., Stephensen, J., Hotton, P., & Zwi, K. (2023). Developmental trajectories of socio-emotional outcomes of children and young people in out-of-home care–Insights from data of Pathways of Care Longitudinal Study (POCLS). *Child Abuse & Neglect*, 106196.

Huang, C. X., Halfon, N., Sastry, N., Chung, P. J., & Schickedanz, A. (2023, Jul 1). Positive Childhood Experiences and Adult Health Outcomes. *Pediatrics, 152*(1). https://doi.org/10.1542/peds.2022-060951

Huibregtse, B., Corley, R., Wadsworth, S., & Stallings, M. (2012). Change in heritability of substance use behavior through development: Exploring the longitudinal role of shared environment [Conference Abstract]. *Behavior Genetics, 42*(6), 940. https://doi.org/10.1007/s10519-012-9566-6

Hunt, E. T., Armstrong, B., Beets, M. W., Turner-McGrievy, G., & Weaver, R. G. (2023). Interpersonal and Environmental Protective Factors and Their Associations With Children’s Weight Status. *Journal of Primary Care & Community Health, 14*, 21501319231182304.

Jester, J. M., Cranford, J. A., Buu, A., & Zucker, R. A. (2014). Comparing predictors of speeding vs alcohol offense convictions in a high-risk sample [Conference Abstract]. *Alcoholism: Clinical and Experimental Research, 38*, 200A. https://www.embase.com/search/results?subaction=viewrecord&id=L71503883&from=export

Jones, E. J., Font, S. A., & Schreier, H. M. C. (2019). Child maltreatment, protective factors during adolescence, and adult physical and psychological well-being [Conference Abstract]. *Psychosomatic Medicine, 81*(4), A93. https://doi.org/10.1097/PSY.0000000000000699

Kaman, A., Otto, C., Klasen, F., Westenhöfer, J., Reiss, F., Hölling, H., & Ravens-Sieberer, U. (2021, Feb 1). Risk and resource factors for depressive symptoms during adolescence and emerging adulthood - A 5-year follow-up using population-based data of the BELLA study. *J Affect Disord, 280*(Pt A), 258-266. https://doi.org/10.1016/j.jad.2020.11.036

Katainen, S., Räikkönen, K., & Keltikangas-Järvinen, L. (1999). Adolescent temperament, perceived social support, and depressive tendencies as predictors of depressive tendencies in young adulthood [Article]. *European Journal of Personality, 13*(3), 183-207. https://doi.org/10.1002/(sici)1099-0984(199905/06)13:3<183::aid-per327>3.0.co;2-z

Koshy, B., Srinivasan, M., Gopalakrishnan, S., Mohan, V. R., Scharf, R., John, S., Beulah, R., Muliyil, J., & Kang, G. (2022, Nov 9). Early Childhood Stimulating Environment Predicts Later Childhood Resilience in an Indian Longitudinal Birth Cohort Study. *Children (Basel), 9*(11). https://doi.org/10.3390/children9111721

Kosterman, R., Mason, W. A., Haggerty, K. P., Hawkins, J. D., Spoth, R., & Redmond, C. (2011, Aug). Positive childhood experiences and positive adult functioning: prosocial continuity and the role of adolescent substance use. *J Adolesc Health, 49*(2), 180-186. https://doi.org/10.1016/j.jadohealth.2010.11.244

La Charite, J., Khan, M., Dudovitz, R., Nuckols, T., Sastry, N., Huang, C., Lei, Y., & Schickedanz, A. (2023, Dec). Specific domains of positive childhood experiences (PCEs) associated with improved adult health: A nationally representative study. *SSM Popul Health, 24*, 101558. https://doi.org/10.1016/j.ssmph.2023.101558

Lee, H., & Schafer, M. (2021, Mar). Are Positive Childhood Experiences Linked to Better Cognitive Functioning in Later Life?: Examining the Role of Life Course Pathways. *J Aging Health, 33*(3-4), 217-226. https://doi.org/10.1177/0898264320972547

Lee, L. O., Aldwin, C. M., Kubzansky, L. D., Chen, E., Mroczek, D. K., Wang, J. M., & Spiro, A. (2015, Dec). Do cherished children age successfully? Longitudinal findings from the Veterans Affairs Normative Aging Study. *Psychol Aging, 30*(4), 894-910. https://doi.org/10.1037/pag0000050

Lyu, J., & Agrigoroaei, S. (2017). Childhood misfortune and adult health in a national study: The mediational role of the quality of social relations. *The International Journal of Aging & Human Development, 84*(3), 213-230. https://doi.org/10.1177/0091415016669147

Mahatmya, D. (2012). *Pathways to positive youth development: Identifying family, school, and neighborhood influences on civic involvement in emerging adulthood* ProQuest Information & Learning]. psyh. https://search.ebscohost.com/login.aspx?direct=true&db=psyh&AN=2012-99060-426&site=ehost-live

Mason, S. M., Frazier, P. A., Renner, L. M., Fulkerson, J. A., & Rich-Edwards, J. W. (2022, Jan). Childhood Abuse-Related Weight Gain: An Investigation of Potential Resilience Factors. *Am J Prev Med, 62*(1), 77-86. https://doi.org/10.1016/j.amepre.2021.07.011

Meinck, F., Orkin, M., & Cluver, L. (2021, Nov 11). Accelerating Sustainable Development Goals for South African adolescents from high HIV prevalence areas: a longitudinal path analysis. *BMC Med, 19*(1), 263. https://doi.org/10.1186/s12916-021-02137-8

Orbuch, R., Sheehan, K., Rosenow, W., & Yousuf, S. (2021). Childhood protective factors and adult health outcomes [Conference Abstract]. *Pediatrics, 147*(3), 196-197. https://doi.org/10.1542/peds.147.3_MeetingAbstract.196-a

Reynolds, A. J., & Ou, S. R. (2011, Mar-Apr). Paths of effects from preschool to adult well-being: a confirmatory analysis of the child-parent center program. *Child Dev, 82*(2), 555-582. https://doi.org/10.1111/j.1467-8624.2010.01562.x

Skodol, A. E., Bender, D. S., Pagano, M. E., Shea, M. T., Yen, S., Sanislow, C. A., Grilo, C. M., Daversa, M. T., Stout, R. L., Zanarini, M. C., McGlashan, T. H., & Gunderson, J. G. (2007, Jul). Positive childhood experiences: resilience and recovery from personality disorder in early adulthood. *J Clin Psychiatry, 68*(7), 1102-1108. https://doi.org/10.4088/jcp.v68n0719

Slopen, N., Chen, Y., Guida, J. L., Albert, M. A., & Williams, D. R. (2017, Apr). Positive childhood experiences and ideal cardiovascular health in midlife: Associations and mediators. *Prev Med, 97*, 72-79. https://doi.org/10.1016/j.ypmed.2017.01.002

Sonuga-Barke, E. J. S., Kennedy, M., Kumsta, R., Knights, N., Golm, D., Rutter, M., Maughan, B., Schlotz, W., & Kreppner, J. (2017, Apr 15). Child-to-adult neurodevelopmental and mental health trajectories after early life deprivation: the young adult follow-up of the longitudinal English and Romanian Adoptees study. *Lancet, 389*(10078), 1539-1548. https://doi.org/10.1016/s0140-6736(17)30045-4

Thomann Mitchell, E. (2014). *Difficulties among children exposed to multiple risks and the protective role of social support* ProQuest Information & Learning]. psyh. https://search.ebscohost.com/login.aspx?direct=true&db=psyh&AN=2014-99170-569&site=ehost-live

Topitzes, J., Mersky, J. P., & Reynolds, A. J. (2010, Jun). Child maltreatment and adult cigarette smoking: a long-term developmental model. *J Pediatr Psychol, 35*(5), 484-498. https://doi.org/10.1093/jpepsy/jsp119

Trejos-Castillo, E., & Martinez, J. (2011). Does acculturation equal A+? Correlates of college-going expectations amongst Hispanic and Asian immigrant youth. In *Acculturation: Implications for Individuals, Families and Societies* (pp. 167-184). https://www.scopus.com/inward/record.uri?eid=2-s2.0-84891980742&partnerID=40&md5=18fb9e4fd30ccbd7370505b202b81d31

Triolo, F., Sjöberg, L., Vetrano, D. L., Darin-Mattsson, A., Bertolotti, M., Fratiglioni, L., & Dekhtyar, S. (2019). Early-life financial strain, social engagement and old age depressive symptoms: A population-based study [Conference Abstract]. *European Geriatric Medicine, 10*, S29. https://doi.org/10.1007/s41999-019-00221-0

Triolo, F., Sjöberg, L., Vetrano, D. L., Darin-Mattsson, A., Bertolotti, M., Fratiglioni, L., & Dekhtyar, S. (2020, Feb 15). Social engagement in late life may attenuate the burden of depressive symptoms due to financial strain in childhood. *J Affect Disord, 263*, 336-343. https://doi.org/10.1016/j.jad.2019.11.163

van Delft, I., Finkenauer, C., & Verbruggen, J. (2016, May). Child Maltreatment and Social Connectedness Among Formerly Institutionalized Females: Links With Depression. *J Interpers Violence, 31*(8), 1393-1412. https://doi.org/10.1177/0886260514567959

van Harmelen, A. L., Kievit, R. A., Ioannidis, K., Neufeld, S., Jones, P. B., Bullmore, E., Dolan, R., Fonagy, P., & Goodyer, I. (2017, Oct). Adolescent friendships predict later resilient functioning across psychosocial domains in a healthy community cohort. *Psychol Med, 47*(13), 2312-2322. https://doi.org/10.1017/s0033291717000836

Wang, D., Jiang, Q., Yang, Z., & Choi, J. K. (2021, Sep 1). The longitudinal influences of adverse childhood experiences and positive childhood experiences at family, school, and neighborhood on adolescent depression and anxiety. *J Affect Disord, 292*, 542-551. https://doi.org/10.1016/j.jad.2021.05.108

Williams, A. L., & Merten, M. J. (2015). Characteristics of early community adversity, social resources, and adolescent long-term mental health [Article]. *Journal of Community Psychology, 43*(2), 125-141. https://doi.org/10.1002/jcop.21669

Zhang, L., Fang, J., Zhang, D., Wan, Y., Gong, C., Su, P., Tao, F., & Sun, Y. (2021, Mar 1). Poly-victimization and psychopathological symptoms in adolescence: Examining the potential buffering effect of positive childhood experiences. *J Affect Disord, 282*, 1308-1314. https://doi.org/10.1016/j.jad.2021.01.011

Zhu, N., Hawke, L. D., Sanches, M. R., & Henderson, J. (2023, Apr). The impact of child maltreatment on mental health and substance use trajectories among adolescents. *Early Interv Psychiatry, 17*(4), 394-403. https://doi.org/10.1111/eip.13339
